# Single-session tDCS over the dominant hemisphere affects contralateral spectral EEG power, but does not enhance neurofeedback-guided event-related desynchronization of the non-dominant hemisphere's sensorimotor rhythm

**DOI:** 10.1371/journal.pone.0193004

**Published:** 2018-03-07

**Authors:** Valeria Mondini, Anna Lisa Mangia, Angelo Cappello

**Affiliations:** Department of Electrical, Electronic and Information Engineering (DEI), University of Bologna, Cesena, Italy; University Medical Center Goettingen, GERMANY

## Abstract

**Background and objective:**

Transcranial direct current stimulation (tDCS) and neurofeedback-guided motor imagery (MI) have attracted considerable interest in neurorehabilitation, given their ability to influence neuroplasticity. As tDCS has been shown to modulate event-related desynchronization (ERD), the neural signature of motor imagery detected for neurofeedback, a combination of the techniques was recently proposed. One limitation of this approach is that the area targeted for stimulation is the same from which the signal for neurofeedback is acquired. As tDCS may interfere with proximal electroencephalographic (EEG) electrodes, in this study our aim was to test whether contralateral tDCS could have interhemispheric effects on the spectral power of the unstimulated hemisphere, possibly mediated by transcallosal connection, and whether such effects could be used to enhance ERD magnitudes. A contralateral stimulation approach would indeed facilitate co-registration, as the stimulation electrode would be far from the recording sites.

**Methods:**

Twenty right-handed healthy volunteers (aged 21 to 32) participated in the study: ten assigned to cathodal, ten to anodal versus sham stimulation. We applied stimulation over the dominant (left) hemisphere, and assessed ERD and spectral power over the non-dominant (right) hemisphere. The effect of tDCS was evaluated over time. Spectral power was assessed in theta, alpha and beta bands, under both rest and MI conditions, while ERD was evaluated in alpha and beta bands.

**Results:**

Two main findings emerged: (1) contralateral alpha-ERD was reduced after anodal (*p* = 0.0147), but not enhanced after cathodal tDCS; (2) both stimulations had remote effects on the spectral power of the contralateral hemisphere, particularly in theta and alpha (significant differences in the topographical t-value maps).

**Conclusion:**

The absence of contralateral cathodal ERD enhancement suggests that the protocol is not applicable in the context of MI training. Nevertheless, ERD results of anodal and spectral power results of both stimulations complement recent findings on the distant tDCS effects between functionally related areas.

## 1 Introduction

Over the years, transcranial direct current stimulation (tDCS) and mental practice in the form of motor imagery (MI) have attracted considerable interest with regard to neurorehabilitation, for example in stroke patients [1]. Both techniques can indeed promote neuroplasticity, thus boosting recovery when paired with a standard rehabilitation protocol [1].

tDCS is a noninvasive brain stimulation technique that consists of delivering a low-intensity direct current (usually 1-2mA in 35cm^2^ electrodes) for a limited amount of time (10-20 minutes), through a pair of electrodes, at least one of which is placed on the scalp [2]. It is well established that tDCS induces polarity-dependent excitability modulations, with anodal stimulation increasing and cathodal stimulation decreasing cortical excitability [3]. The modulation of cortical excitability influences neuroplasticity, which can eventually enhance motor recovery [1,4,5].

As regards motor imagery (MI), its use in neurorehabilitation was proposed given the technique’s ability to recruit approximately the same areas as overt movement, regardless of the residual level of motor control [1,6,7]. The reiterated engagement of the motor system induced by MI training is designed to promote the neuroplasticity of the area, thus enhancing recovery [1,6,7]. However, as motor imagery is a purely mental task, it has recently been shown that a better rehabilitation outcome can be achieved when the practice is guided by a dedicated Brain-Computer Interface (BCI) [6], and a neurofeedback system in particular, as this closes the loop by providing appropriate feedback to the user.

Generally speaking, a BCI is a system that records neural activity and translates it into a control signal for a particular device (e.g. robotic arm, machine, computer) [8]. In addition to being used for communication or control purposes [9], BCIs have recently emerged in the context of neurorehabilitation also [7,10,11], where they are employed to decode the neurophysiological features associated with attempted movements or motor imagery to give feedback to the user accordingly (neurofeedback). At a cortical level, the neural signature of motor imagery is the event-related desynchronization (ERD) of sensorimotor rhythms (SMR) in the motor area contralateral to the movement [12,13]. By detecting the ERD and providing contingent feedback to the patient, the BCI objectifies the motor network engagement and encourages the desired modulation of cortical rhythms, thus guiding the practice while keeping the user engaged and motivated [6,7].

Even though tDCS and neurofeedback-guided MI training are usually employed independently of each other, a combination of the two has recently been suggested [14–18]. The purpose of the combination would be to produce an ERD enhancement by means of tDCS, to facilitate BCI control [14–18].

Several recent studies have shown that tDCS can modulate the motor imagery-induced ERD [14–16,18–23]. Most of them agree that anodal stimulation increases the strength of the ERD in the stimulated area [14–16,18–20], while cathodal stimulation decreases it [19,20]. It was therefore suggested that anodal tDCS could be used as a conditioning tool to enhance neurofeedback-guided MI training [14–16,18,20].

One limitation of the approach described above is that the target area for stimulation is the same from which the ERD should be recorded to provide neurofeedback. However, since tDCS may induce artifacts in the EEG locations proximal to the stimulation electrode [17,24], it is not possible, at least with a traditional 2-channel tDCS device, to perform neurofeedback training *during* stimulation, unless the stimulation electrode is placed in a non-optimal site, which could decrease the efficacy of tDCS [17]. This situation is typically solved by performing tDCS stimulation first, followed by neurofeedback training [14–16,19,25]. However, provided that the tDCS after-effects are sustained for a limited amount of time [26], this necessitates starting neurofeedback as quickly as possible right after stimulation. Furthermore, it has been suggested that the timing of stimulation is important for motor skill learning [27]. If it were possible to find an experimental setup to facilitate EEG-tDCS co-registration, e.g. by placing the stimulation electrode far enough from the recording ones, this would simplify the application of tDCS to neurofeedback training in both the research and clinical contexts. This would also allow characterizing the tDCS effects not only after but also during stimulation, as in [28].

As a first aim, we investigated the feasibility of an approach where tDCS is applied during neurofeedback-guided MI training not to the ipsilateral, but to the contralateral motor cortex. This would allow simplifying the experimental setup, as the stimulation electrode would be placed far from the EEG recording sites. Our idea was to test whether it was possible to produce an enhancement of the motor imagery-induced ERD on the target motor cortex by exploiting the phenomenon of interhemispheric inhibition [29]. We hypothesize that tDCS may influence the ERD on the contralateral motor cortex with an opposite sign with respect to the ipsilateral modulation, i.e. that cathodal stimulation would bring about facilitation while anodal stimulation would produce an inhibition of contralateral ERDs. Since this is the first time contralateral tDCS is examined with regard to its rehabilitative potential, we applied our protocol to healthy users.

In addition to testing the feasibility of our approach to enhance neurofeedback-guided MI training, we also aimed at clarifying the remote effects of tDCS on contralateral EEG rhythms. Indeed, there are only a few studies that marginally address this issue, particularly during motor imagery, and their results are not fully consistent or comparable given the different experimental setups [15,21–23,30]. In Notturno et al. [30], where the local and remote effects of tDCS were evaluated during a finger tapping task, an increase in bilateral ERD in the alpha band was observed after anodal stimulation, while there were no effects caused by cathodal or sham (i.e. unreal) stimulation. In Lapenta et al. [21], an opposing effect of tDCS between hemispheres emerged over the ERDs induced by motor imagery. Conversely, in Wei et al [15], anodal stimulation of the right motor cortex increased the ERD of both left (ipsilateral) and right (contralateral) hand motor imagery. Finally, Roy et al. [22] and Baxter et al. [23] did not find stimulation to have an effect on contralateral ERDs.

In regard to the first aim of this study, i.e. testing the feasibility of contralateral tDCS for neurorehabilitation, we hypothesized a situation of single-hand motor imagery training, guided by a dedicated neurofeedback system. The motor imagery task was repeated before, during, and after stimulation, so that the contralateral tDCS effects could be characterized over time. In regard to the second, more explorative nature of the study, we completed the ERD analysis with a study of EEG spectral power under “reference" or “motor imagery” conditions. By providing a complete analysis of both ERD and power over time, we believe that this work will contribute to the understanding and characterization of the distant tDCS effects on cortical rhythms.

## 2 Material and methods

### 2.1 Participants

Twenty healthy volunteers (aged 21 to 32, median 26, nine males) took part in the study. Since both interhemispheric connections and the modulating effects of tDCS are influenced by handedness [31–33], we only enrolled right-handed volunteers, as assessed by the Edinburgh Handedness Inventory [34]. The study conformed to the Declaration of Helsinki and was approved by the Bioethics Committee of the University of Bologna. All participants provided written consent to participate in the study.

### 2.2 tDCS stimulation

We tested both anodal and cathodal stimulations in a sham-controlled design, so each volunteer participated in two rounds of experiments, alternatively receiving real or sham stimulation. Ten out of twenty subjects received cathodal versus sham stimulation, while the other ten underwent anodal versus sham. All participants were blinded to their stimulation condition. The two rounds of experiments were separated by at least 24 hours, but they were always completed within a week (with a median value of the interval of 2 days). The order of real and sham stimulations was randomized and counterbalanced in each group, to compensate for the learning effect when averaging across subjects.

tDCS was delivered by a battery-driven, constant-current stimulator (neuroConn GmbH, Ehrenbergstr, Ilmenau, Germany) through a pair of round, water-soaked sponge electrodes (16 cm^2^). We set a current intensity of 0.7mA and a stimulation time of 15 minutes, with 20s ramp up and 25s ramp down in addition. Under the sham condition, the current was supplied for only 60s (20s ramp up, 15s of stimulation and 25s ramp down), just to mimic the physical tingling sensation at the beginning of stimulation.

The selected stimulation parameters are justified in the following lines. We chose a stimulation time of 15 minutes to make sure that tDCS effects outlasted the end of stimulation for a sufficient time to complete the experiment. Over 11 minutes of stimulation duration, the after-effects should indeed remain for approximately 1 hour [3]. In regard to stimulation intensity, previous research showed that a current of 1 mA in 35cm^2^ can effectively modulate cortical excitability [3,26]. Given the smaller area of our electrodes (16cm^2^) and the fact that we were testing an indirect type of stimulation (i.e. contralateral), we decided to apply a current leading to a slightly higher current density (0.0437mA/cm^2^), equivalent to 1.5mA in standard 35cm^2^ electrodes.

We designed the experiment so that the dominant hemisphere was stimulated, therefore we placed the active electrode (the anode in anodal and the cathode in cathodal stimulation) over the left motor cortex and the reference over the right supraorbital region, as this montage was shown to be optimal for enhancing motor cortex excitability [3,35]. The stimulation sites (C3 and Fp2) were determined according to the international 10-20 system, as previous studies have confirmed the correspondence between C3, C4 and the primary motor cortices [36,37].

### 2.3 EEG recording

**Fig 1:**
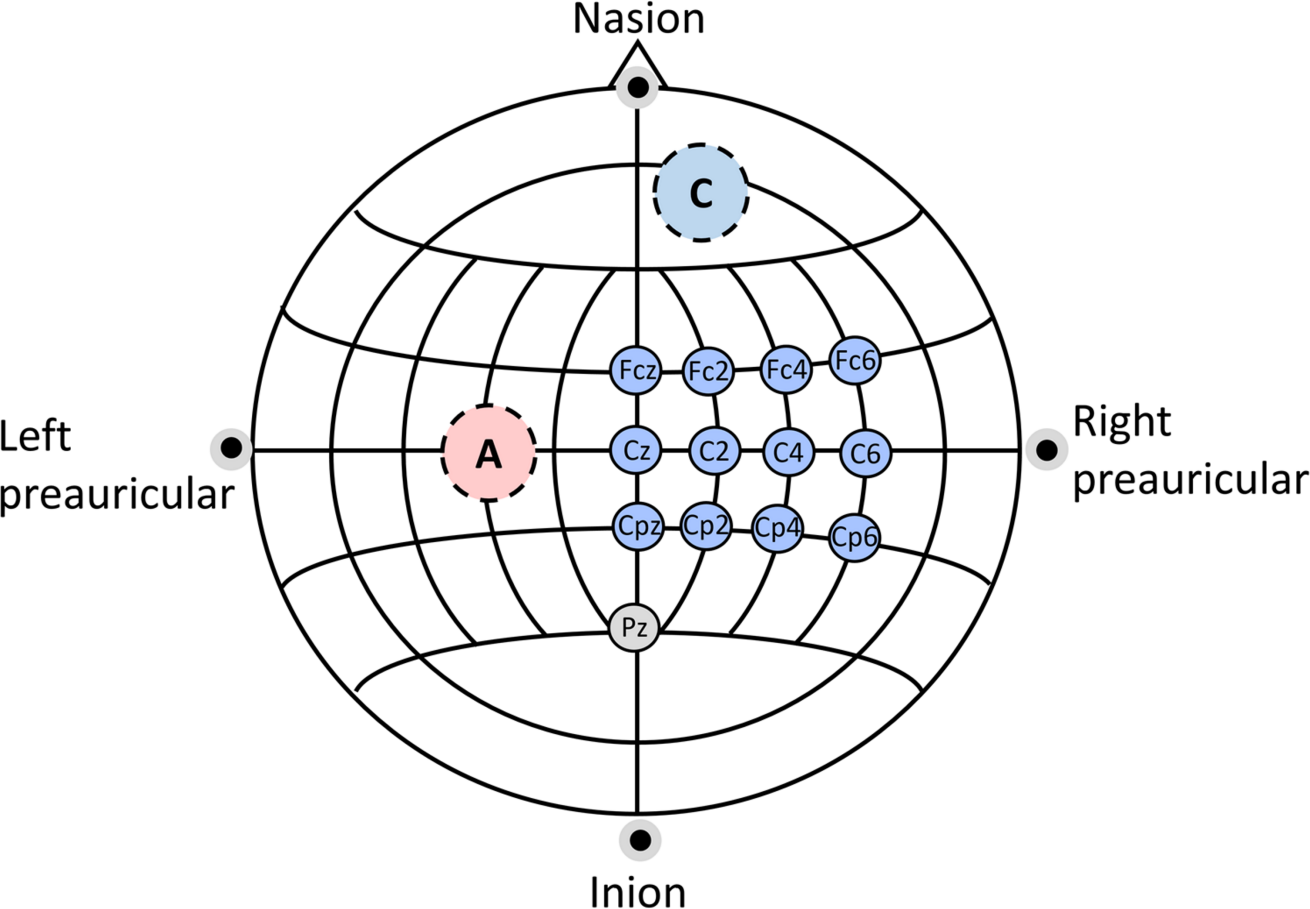
EEG recording sites. Location of the twelve EEG recording sites (right hemisphere), the ground electrode (Pz) and the two stimulation electrodes (anodal stimulation in this example).

We recorded the EEG signals using a Brainbox EEG-1166 amplifier, with a 128 Hz sampling frequency and according to the extended international 10-20 layout [38]. We acquired twelve passive wet electrodes overlying the right motor cortex (Fcz, Fc2, Fc4, Fc6, Cz, C2, C4, C6, Cpz, Cp2, Cp4, Cp6), with an additional ground electrode in Pz. After being recorded with respect to ground (Pz), the electrodes were re-referenced to their common average reference and used for both online neurofeedback operation and offline analyses. An outline of the experimental setup is shown in Fig 1.

### 2.4 Experimental paradigm

During the experiments, participants were seated in a comfortable chair in front of the pc screen running the neurofeedback system software. They were asked to keep their gaze fixed, their muscles relaxed and their eyes open.

To evaluate the effects of tDCS on ERD, each participant performed the motor imagery task (left hand motor imagery) before, during and after stimulation. All participants underwent two days of experiments, to compare real and sham stimulation conditions.

The execution of the motor imagery task was timed and guided by the neurofeedback software, which gave feedback in a cue-paced paradigm (see section 2.5 for details). The features controlling the feedback were selected for each participant through a short calibration phase, which preceded the first experimental day.

**Fig 2:**
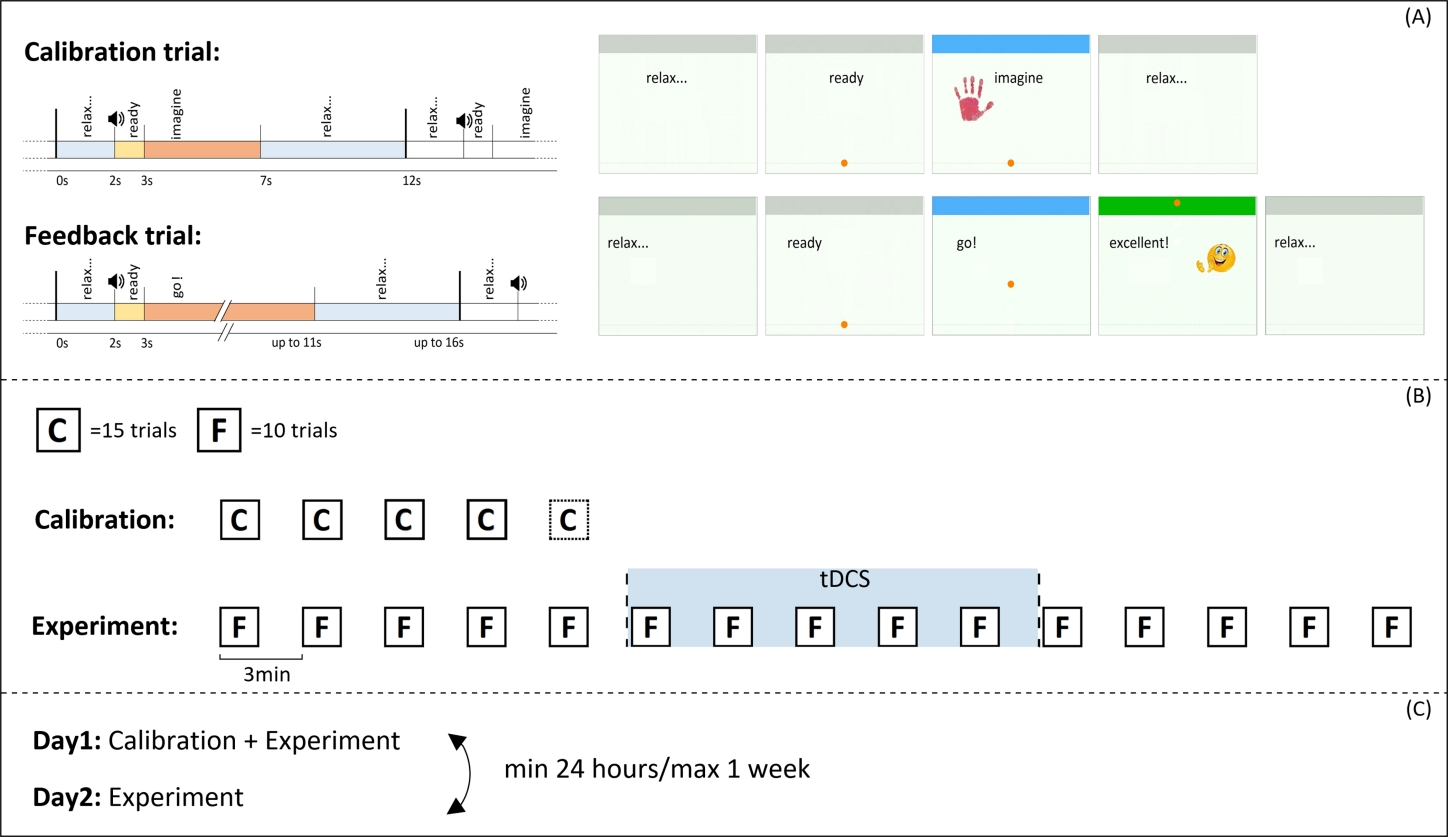
The experimental paradigm. Outline of the experimental paradigm at different levels of detail. In (A) the structure of calibration and feedback trials is recalled, while in (B) and (C) we detail the structure of runs and the composition of the experimental days.

The execution of the neurofeedback software was organized into runs, and the runs into trials. The calibration step consisted of four or five runs, depending on the number of rejected trials (see section 2.5.1), while the experiment consisted of fifteen runs of neurofeedback online operation: five runs before, five during and five immediately after the tDCS stimulation. The runs were repeated precisely every 3 minutes, so that they were always aligned both with the onset/offset time of stimulation and across subjects (see Fig 2 for an outline of the experimental paradigm).

### 2.5 The neurofeedback software

We conducted the experiments using custom neurofeedback software, specifically developed for the study. The software, based on LabVIEW (National Instruments) and MATLAB (the MathWorks, Inc), was inspired by the system presented in [6,39], given its efficacy in guiding MI training in stroke patients [6].

The software displayed visual feedback, encoded in a one-dimensional cursor movement, where the speed and direction of the cursor were given proportionally to the instantaneous ERD. Subjects were assigned the goal of reaching the top of the screen in the shortest possible time (i.e. to produce a strong ERD), or at least to keep the cursor on a stable direction towards the top (i.e. to produce a stable desynchronization). In order to encourage the spontaneous desynchronization pattern of the user, the ERD was computed using the pair of subject-specific locations and frequency bands that best showed SMR modulation. The software therefore included two modules: one for calibration (without feedback), and one for online operation (with feedback). The two modules are detailed in the following subsections.

Both calibration and neurofeedback were organized into runs, and the runs into trials. The calibration and neurofeedback runs consisted of fifteen and ten trials, respectively.

Each trial began with the word “relax” appearing on the screen. After 2 seconds, the word “ready” was displayed, together with a warning tone and the appearance of the cursor at the bottom of the screen. Starting from second 3, the subject was asked to perform the MI task (left hand grasping) for a fixed time of 4s during system calibration (no feedback) and until the cursor reached the top (or with an 8s timeout, after which the cursor disappeared, see section 2.5.2) during online operation. The trials ended with 5 more seconds of rest (Fig 2).

#### 2.5.1 Calibration

The features controlling cursor motion, the visual feedback, were selected through a short calibration phase. The calibration module included an automated artifact rejection algorithm, implemented as in [40]. The algorithm marked the trials as outliers if their 25-40Hz power in the active period (0-7s) was higher than three standard deviations from the grand mean of this condition. The algorithm iteratively recomputed both the grand mean and the standard deviation after each outlier rejection, and stopped when no more trials matched the condition to be rejected [40].

After each calibration run, the system displayed the total number of rejected trials, therefore it was possible to evaluate if the remaining ones were sufficient for feature selection. As soon as fifty clean trials were collected, the software launched an executable MATLAB file to perform the offline analysis of the data. The aim of this analysis was to find the pair of contiguous channels and frequency bins that showed the spontaneous SMR modulation of the user.

**Fig 3:**
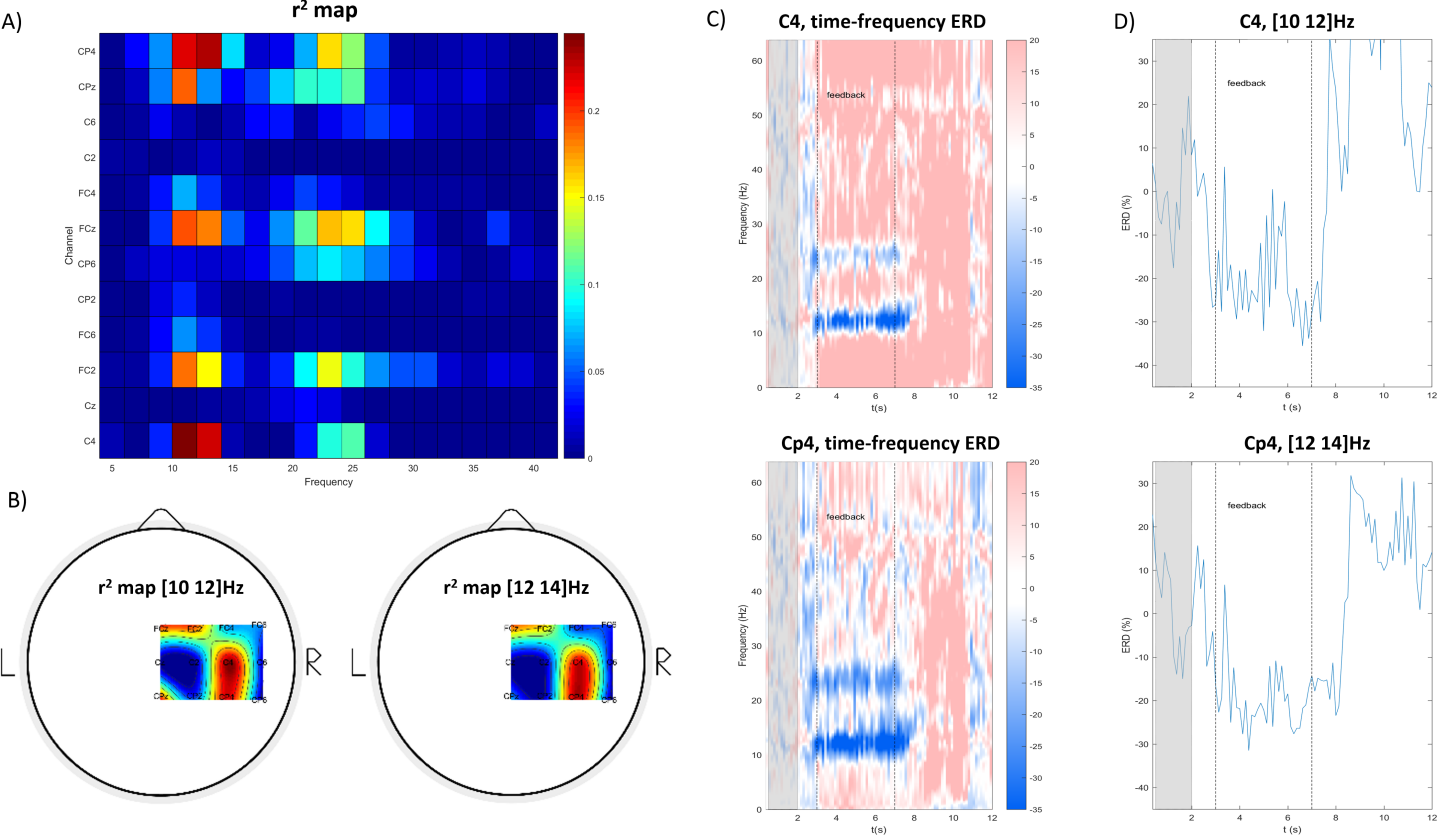
An example of the selection of subject-specific channels and frequency bands. In (A) and (B), an example of the matricial and topographical arrangements of r^2^ values computed from calibration trials is shown. In (C) and (D), the candidate channels (C4 and Cp4) and frequency bands (10-12 Hz and 12-14 Hz) are investigated further using time-frequency transforms (C)) and ERD time courses (D)).

Feature selection was accomplished similarly to [6,39]. After segmenting the trials into overlapping 1s epochs by shifting a 1s-Blackman-Harris-window by 0.125s, we computed the power spectrum by means of a modified periodogram, and extracted from each epoch the power values in the 8-30Hz range with 2Hz bins. We considered 8-30Hz to be a reasonable range for SMR modulation, and the choice was inspired by the work in [6], where feedback features covered both the alpha and the beta bands. We labeled as “reference” the epochs in the 0-2s trial period and as “MI” the ones in the 3-7s period. In order to highlight the most discriminating features of the two conditions, the determination coefficient *r*^*2*^ was computed for every channel and frequency bin, as in [39,41]. We compiled the *r*^*2*^ values both in a channel-frequency matrix and in topographical scalp maps, which made it possible to visually identify the locations and bands with the highest SMR modulation (see Fig 3 for an example). The candidate locations were investigated further through time-frequency analyses and ERD time-courses, both averaged across trials. The two locations and frequency bins that best showed the spontaneous ERD were manually selected after visualization. We always selected a pair of contiguous electrodes and, if different, contiguous frequency bins. The ERDs in the selected channels would be linearly combined to form a single control signal. The weights of the linear combination were also determined manually during calibration. To guarantee better protection against the repositioning of the EEG cap between experimental days, we always tried to choose similar or identical weights for the two locations. If the ERD was significantly more evident in one of the two selected locations, we imbalanced the coefficients up to 0.6 and 0.4 in favor of the channel showing the stronger ERD. The electrodes, bands and weights chosen for each participant are reported in S1 Table.

#### 2.5.2 Neurofeedback (online operation)

During online operation, participants received visual feedback during motor imagery: a cursor appeared on the screen and started moving with speed and direction proportional to the ERDs composing the control signal.

The ERD at a certain frequency was computed as follows:

$$ERD(f,t)=\frac{P(f,t)-Pref(f)}{Pref(f)}$$ (1)

where *P(f*,*t)* is the power in the current time-point and *Pref(f)* is the average power in the reference period (1.5s-interval before the word “ready”). During online operation, the power spectrum was extracted from 0.375s-long time windows every 0.125s, through a Yule-Walker autoregressive (AR) algorithm of order *p* = 16. At each computation, the ERDs in the selected channels and frequency bins were linearly combined to form the control signal, and the cursor position was updated according to the module and sign of the composite ERD. Whenever a negative ERD value was detected (desynchronization), the cursor moved towards the top, while in the case of a positive value (synchronization) the cursor moved downwards. The distance spanned by the cursor was proportional to the ERD absolute value, with a gain that was trialwise adjusted in order to maintain the challenge for the user.

There was a target at the top of the screen that turned green if it was hit, together with the appearance of the word “good!”. If the hit was achieved in less than 2s, or the direction of the cursor was maintained for more than 2s, the word “good!” was replaced by “excellent!”, and an additional smiley face appeared (Fig 2). If the cursor did not reach the top, it simply disappeared after 8s.

### 2.6 Offline analyses

We performed offline analyses with custom scripts using MATLAB (The MathWorks, Inc) and EEGLAB toolbox [42].

We extracted three outcome measures from each subject and trial: i) the ERD values, and the spectral power values in the ii) “reference” or iii) “MI” task condition.

For all analyses, signals were zero-phase band-pass filtered (using a Butterworth filter of order ten) in the 1-40Hz band and re-referenced to their common average reference. We visually checked all trials, inspecting both their timecourse and their spectrum, and excluded those containing muscular or movement artifacts from further investigations.

To account for the between-subject variability in the alpha peak, we determined the Individual Alpha Frequency (IAF) of each participant [43]. Similarly to [6], we consequently defined four IAF-based frequency bands: theta (from IAF-6Hz to IAF-2Hz), alpha (IAF-2Hz to IAF+2Hz), and two beta bands, beta_low_ (IAF+2Hz to IAF+11Hz) and beta_high_ (IAF+11Hz to IAF+20Hz).

We extracted the ERD values for each subject and trial by considering the same weights and locations as in the control signal. Power spectral analysis was performed as online, i.e. by means of Yule-Walker autoregressive (AR) algorithm of order *p* = 16 on 0.375s-long time windows every 0.125s. For each time-window, the ERDs in the selected channels were evaluated according to equation (1), and then linearly combined. The minimum value of the composite signal was considered as the output ERD value for the trial. The ERDs were evaluated in the three frequency bands typical for SMR modulation: alpha, beta_low_ and beta_high_.

In regard to power spectral analysis, we extracted from each trial the average values of power in the “reference” (0-2s period) or “MI” condition (feedback period of the trial). Differently from ERDs, the analysis was accomplished by means of a modified periodogram, on overlapping 1s epochs by shifting a 1s-Blackman-Harris-window by 0.125s. The power was computed for each channel and frequency band (theta, alpha, beta_low_ and beta_high_). In order to compare the data of all subjects, we performed an intra-subject normalization by dividing the power of each band, electrode and trial by the median value of the corresponding power in the pre-stimulation trials (i.e. the median value of the first 5x10 trials, excluding the artifactual ones). The choice of the median instead of the mean was justified by the shape of the power distribution, which was found to be non-normal despite the relatively large sample size.

### 2.7 Statistical analyses

#### 2.7.1 ERD and spectral power

We preliminarily tested for the normality of both spectral power and ERD distributions through a Kolmogorov-Smirnov test [44]. Since we did not find normal distributions, we transformed all data before performing ANOVA analyses, by means of Box-Cox transformations [45]. The lambda λ parameter of the transformation was estimated separately for the families of ERD and spectral power distributions. After transformation, the data were found to be normally distributed.

We performed a multiway ANOVA analysis for both power values and ERDs, taking single trials into account. We repeated the analyses separately for the cathodal and the anodal group, using the data of all subjects in each group. The multiway ANOVA analysis was aimed at investigating the effects of the (between-subject) factors *time* and *stimulation*. However, to comprehensively describe our data and consider all dependencies among samples, we also included the between-subject factors *frequency band*, *subject* and additionally, in the case of spectral power analysis, the within-subject factor *electrode*. The factor *time* had three levels, depending on the condition of trials with respect to stimulation onset: before (runs 1-5, level *pre*), during (runs 6-10, level *during*) or after (runs 11-15, level *post*) stimulation. The factor *stimulation* had two levels, *stim* for actual or *sham* for sham stimulation, depending on which was administered. The factor *frequency band* had three levels in the case of ERD (alpha, beta_low_ and beta_high_) and four levels in the case of spectral power (theta, alpha, beta_low_ and beta_high_), depending on the number of bands considered in the analysis. The factor *subject* had ten levels, one for each subject. In the case of spectral power, the factor *electrode* finally had twelve levels, one for each recorded electrode. The ANOVA analysis tested the existence of significant effects given by the included factors and, in particular, we were interested in significant effects due to i) *time* ii) *stimulation* or iii) interactions between *time* and *stimulation*.

When appropriate, we conducted post-hoc analyses with Bonferroni correction [46]. These tests made it possible to compare, for each band, the sham and stimulation condition at each time point (*pre*-*stim* versus *pre*-*sham*, *during*-*stim* versus *during*-*sham* and *post*-*stim* versus *post*-*sham*). We considered a significance level *p* = 0.025 for all analyses (i.e. a significance of *p* = 0.05 divided by two as the analyses were repeated independently for two groups of subjects, the cathodal and the anodal group).

To allow for a global interpretation of the results on spectral power, we extracted the t-values from post-hoc comparisons between sham and real stimulation conditions and arranged them in topographical scalp maps. We then marked the electrodes corresponding to a *p*<0.025 with a cross (where the p-values were already Bonferroni-corrected for multiple comparisons).

#### 2.7.2 Side-effects questionnaire

Immediately after the experiments, we administered a side-effects questionnaire to each participant to evaluate whether there were differences in their physical perception of tDCS. If no differences between real and sham stimulation are found, this supports the view that tDCS effects over EEG are not due to the physical sensations associated with actual stimulation. The questionnaire asked participants to rate the intensity of the physical perceptions of stimulation on a 1-5 discrete scale. More details on the questionnaire can be found elsewhere [28]. Since the data did not fit a normal distribution, we performed each comparison using the Mann-Whitney U test.

## 3 Results

### 3.1 tDCS effect on ERDs

**Fig 4:**
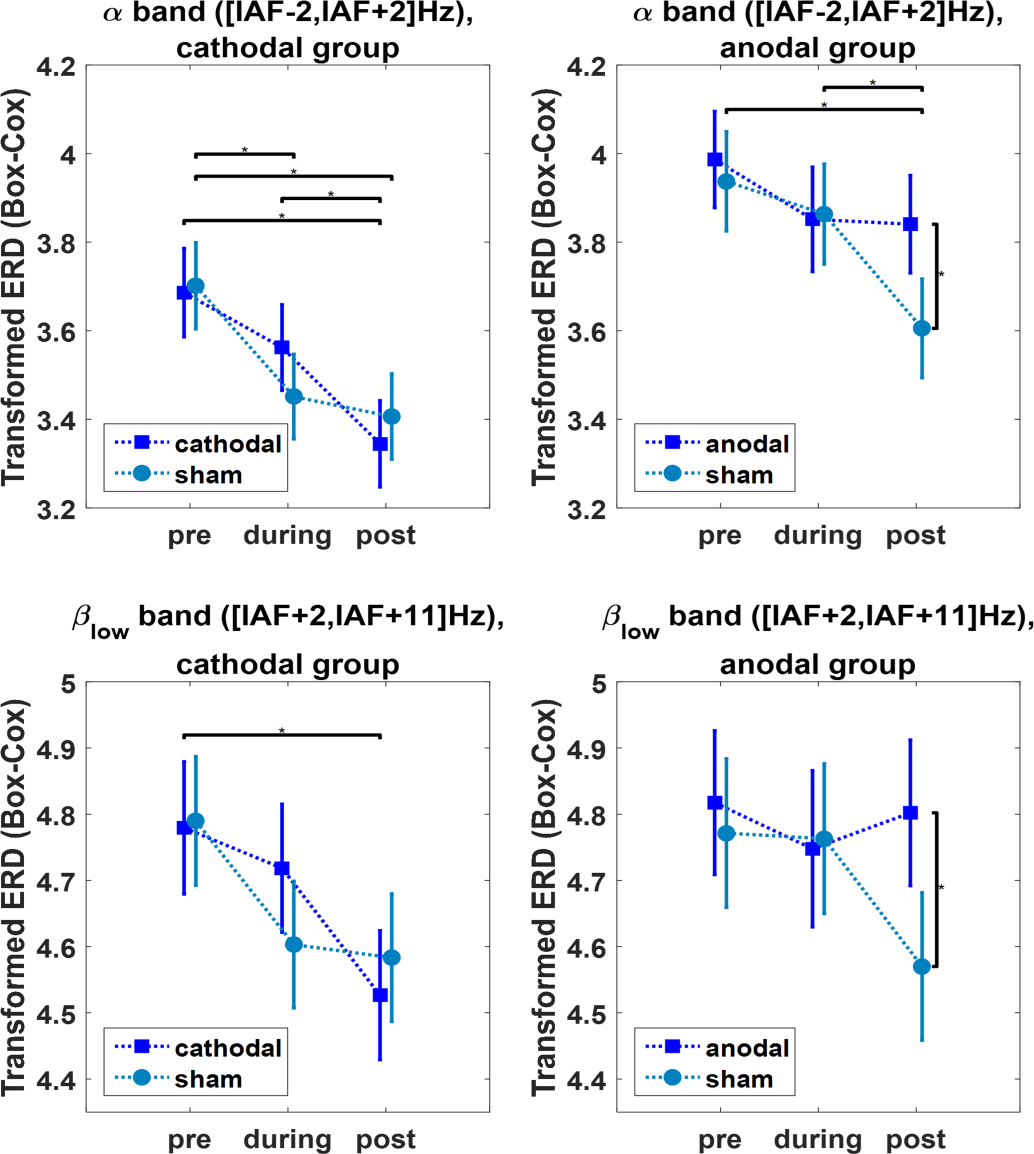
The results on ERDs. The figure shows the mean and confidence intervals (*p* = 0.025, Bonferroni-corrected for multiple comparisons) of the ERDs in all the stimulation-band combinations where significant effects were found. All time points (before, during and after stimulation) and stimulation conditions (real or sham stimulation) are represented. An asterisk (*) marks the statistically different distributions (p<0.025, Bonferroni-corrected), according to post-hoc tests.

We found a significant *time* effect for both the anodal and the cathodal group, with the ERDs being stronger over time on average (cathodal group: F = 32.99, df = 2, *p* = 5·10^-15^, anodal group: F = 9.24, df = 2, *p* = 9·10^-5^). On the contrary, no main *stimulation* effects were found (cathodal group: F = 0.14, df = 1, *p* = 0.71, anodal group: F = 2.45, df = 1, *p* = 0.12), although a significant *time×stimulation* interaction was found for both stimulation groups (cathodal group: F = 4.84, df = 2, *p* = 0.0079, anodal group: F = 7.43, df = 2, *p* = 6·10^-4^). For the sake of completeness, we also report the main results for the factors *frequency band* (cathodal group: F = 1665.7, df = 2, *p*<10^-15^, anodal group: F = 745.6, df = 2, *p*<10^-15^) and *subject* (cathodal group: F = 249.6, df = 9, *p*<10^-15^, anodal group: F = 314.5, df = 9, *p*<10^-15^). The results of the (Bonferroni-corrected) post-hoc comparisons between sham and real stimulations at the different time points are detailed in the following lines and summarized in Fig 4.

In the cathodal group, the magnitude of ERDs tended to increase over time (Fig 4), under both the real and sham stimulation conditions and in every frequency band (alpha, beta_low_ and beta_high_). Notably, in the alpha band *post-stim*, ERDs were significantly stronger both with respect to the *pre-stim* (*p* = 8.9·10^-7^) and to the *during-stim* condition (*p* = 0.024). In addition, *post-sham* ERDs were significantly stronger with respect both to *pre-sham* (*p* = 8.9·10^-5^), as well as *during-sham* with respect to *pre-sham* (*p* = 0.0021). In the beta_low_ band, only the *post-stim* ERDs were significantly stronger than *pre-stim* ERDs (*p* = 0.0025), while other post-hoc comparisons were not statistically significant. In the beta_high_ band, no significant post-hoc differences were revealed.

In the anodal group, the ERDs showed different behavior between the real and sham stimulation conditions overall: while they progressively tended to increase their magnitude in the sham condition (Fig 4), in the real stimulation condition post-stimulation ERDs were not significantly different from pre-stimulation, in each of the tested bands (alpha, beta_low_ and beta_high_). Notably in the *alpha* band, while *post-sham* ERDs were significantly stronger with respect to both *pre-sham* (*p* = 7.7·10^-5^) and *during-sham* (*p* = 0.0173), this behavior was not confirmed in the anodal condition (*post*-*stim* ERDs statistically identical to *pre*-*stim*, *p* = 1). Furthermore, the comparison of real and sham stimulations at each time point revealed that *post*-*sham* ERDs were significantly stronger compared to *post*-*stim* ERDs (*p* = 0.0147), while there was no difference between *pre*-*sham* and *pre*-*stim* conditions (*p* = 1). In the beta_low_ band a behavior similar to *alpha* was found (Fig 4), although the only significant post-hoc comparison was between *post-stim* and *post-sham* ERDs (*p* = 0.0183). Finally, no significant post-hoc differences were found in the beta_high_ band.

### 3.2 tDCS effect on spectral power

**Fig 5:**
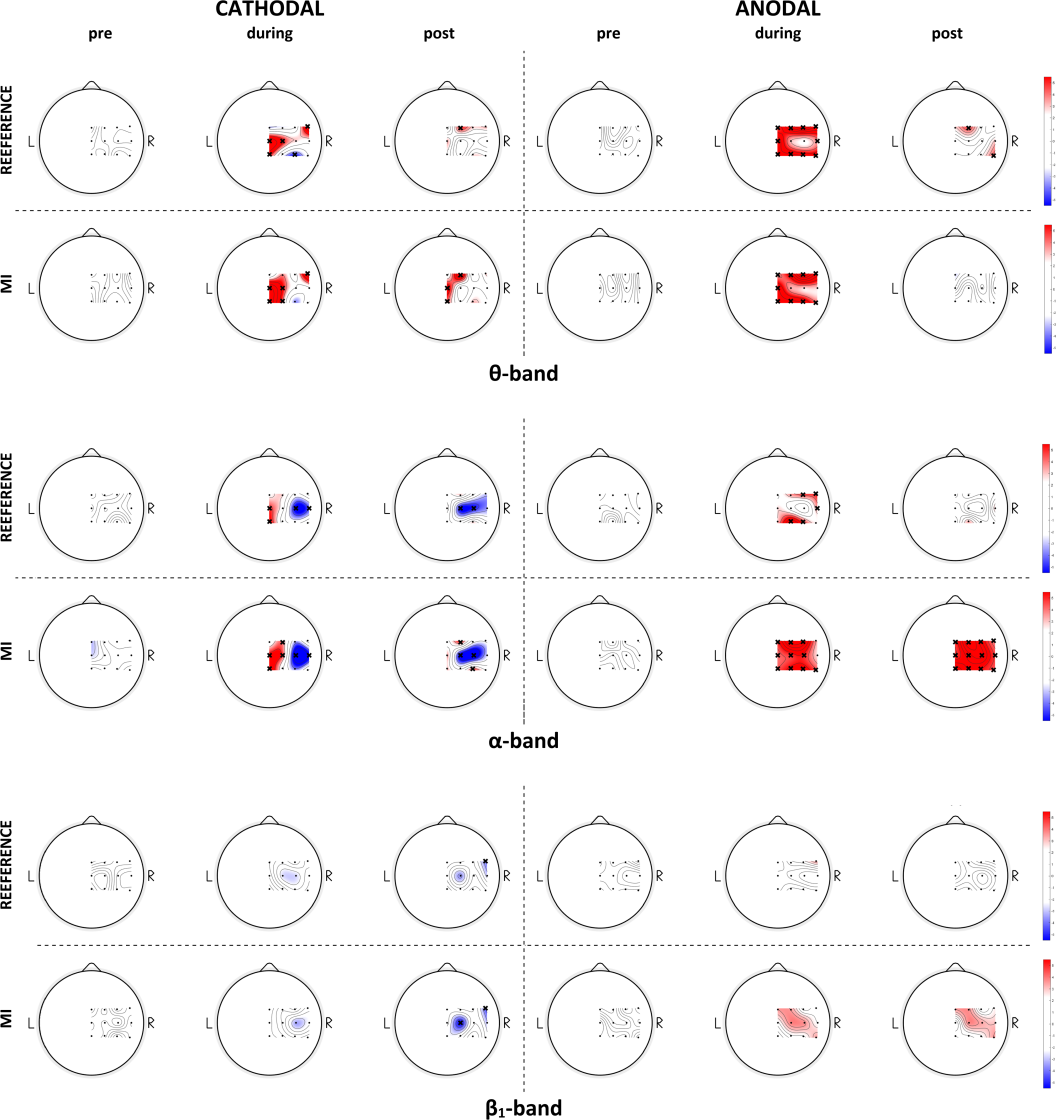
The results of spectral power analysis. Spectral power analysis in the θ (upper), α (center) and β_low_ (lower) band: topographical representations of the t-values from post-hoc comparisons between real and sham stimulation conditions, for each time point (before, during and after stimulation) and task condition (reference or motor imagery). A cross marks the electrodes with statistically significant (p<0.025, Bonferroni-corrected for multiple comparisons) difference.

In regard to spectral power analysis, the detailed results of the ANOVA tests can be found in S2 Table, while the topographical t-value maps from post-hoc comparisons between sham and real stimulations at each time level (pre, during and post-stimulation), for each group (cathodal or anodal) and condition (reference or motor imagery) are reported in Fig 5. In regard to beta bands, Fig 5 only reports the maps relative to beta_low_, as we found approximately the same behavior for beta_low_ and beta_high_.

Overall, spectral power analysis highlighted that tDCS stimulation mainly affected theta and alpha bands, both under rest and motor imagery conditions (Fig 5).

In regard to the theta band, we found a power increase both in the cathodal and in the anodal conditions with respect to sham stimulation, so there was no polarity-specific effect. However, the power increase was limited to the period concomitant with stimulation, while the differences generally vanished in the *post-stimulation* period (Fig 5, upper).

In regard to the alpha band, the analyses revealed both a polarity-specific and, in the anodal group, a task-specific effect. In the cathodal stimulation condition, we found a power decrease with respect to sham in the area around C4, during both rest and motor imagery. In addition, during stimulation the maps highlighted a circumscribed power increase around the central electrodes. In the anodal group, a task-specific effect was also revealed. While in the reference condition, *during*-*anodal* and even more *post*-*anodal* power values were not different from sham, in the motor imagery condition alpha-power significantly increased with respect to sham. Roughly the same behavior appeared in the *during* condition.

In regard to beta bands, we found slighter effects overall compared to theta and alpha. From a qualitative point of view, the behavior of beta power was approximately the same as in alpha, with the only exception being the circumscribed power increase occurring in the central electrodes in the cathodal group.

### 3.3 Side-effects questionnaire

According to the Mann-Whitney U test, we found no differences in the side-effect scores between sham and real stimulations. This supports the view that the tDCS impact on the EEG rhythm is not just a placebo/somatosensory effect due to the physical perception of the stimulation.

## 4 Discussion

The aim of this study was to test whether contralateral tDCS could have interhemispheric effects on the spectral power of the unstimulated hemisphere, and whether such effect could be used to enhance ERD magnitudes in the context of a neurofeedback-guided motor imagery paradigm for neurorehabilitation. Our initial hypothesis was that tDCS could exploit interhemispheric inhibition, i.e. that contralateral cathodal stimulation could result in facilitation of ERD on the unstimulated hemisphere, while contralateral anodal stimulation could result in inhibition.

As discussed more thoroughly in section 4.1, the analysis of ERDs did not confirm our hypothesis, at least in the case of cathodal stimulation: our results indeed suggest that, while contralateral ERDs are reduced during anodal stimulation, there is no symmetric facilitation for cathodal stimulation.

Even though ERD results suggest that contralateral tDCS is not applicable in the context of rehabilitation, spectral power analysis (see section 4.2) revealed the impact of both stimulation polarities on the cortical rhythms of the unstimulated hemisphere. The discussion on spectral power results complements and completes the previous discussion on ERD, suggesting that not only anodal but also cathodal tDCS can impact the rhythms of the unstimulated hemisphere, although the effect is not task-specific.

Finally, section 4.3 discusses the limitations of the study and the generalizability of the findings to a patient population.

### 4.1 tDCS effect on ERDs

Statistical analyses highlighted significant *time* effects and *time×stimulation* interactions in both the anodal and cathodal groups, while no significant *stimulation* effects were found. However, when interpreting ERD results, it should be taken into consideration that the experiments were performed with feedback, so it is reasonable to expect a familiarization effect, both within- and between- sessions. Due to the within-session familiarization, an ERD tendency to increase their magnitude over time could be expected; the between-session effect, on the other hand, might cause the baseline ERD value at the beginning of the second day to be stronger for each subject compared to the first, provided that the participant has undergone an entire neurofeedback training session. In order to smooth the potential bias between baseline ERDs when averaging across subjects, making it possible to highlight the effect of stimulation, we designed the experiment so that the order of real and sham stimulation was counterbalanced within each group. The absence of main *stimulation* effects is encouraging in this sense, as it means that there was not a significant bias between real and sham simulation conditions. Lack of significant differences in every post-hoc comparison of baseline ERDs (*pre-stim* vs *pre-sham*) is further encouraging, and allows us to consider the results of comparisons at other time-levels (*during-stim* vs *during-sham* and *post-stim* vs *post-sham*) more reliable.

Turning now to the significant *time* effect found in both stimulation groups, we suggest it could be easily ascribable to the within-session familiarization, indeed the minimum desynchronization values tended to strengthen over the course of the experiment (Fig 4). However, the significant *time×stimulation* interaction reveals the possibility of an additional role of stimulation. When observing ERD trends in Fig 4, it can be seen that there is one case where the increasing ERD trend is not followed, which is the real stimulation condition in the anodal group, especially in the alpha band. The case referred to is indeed the only one where, while *post-sham* ERDs are significantly stronger than *pre-sham* (so the within-session learning effect is still present in the group in the unreal stimulation condition), *post-stim* ERDs are not different from their baseline condition. We interpret the result by hypothesizing that anodal stimulation might have reduced contralateral ERD and this compensated for the familiarization effect, which did not manifest itself. The significant difference between the *post* conditions of real and sham stimulations further supports this point of view.

We interpret the results as follows: while in the cathodal group ERD behavior is not influenced by stimulation of the homologous contralateral region (indeed both the *stim* and *sham* ERDs show the same increasing tendency), anodal stimulation inhibits the generation of contralateral ERDs, particularly in the alpha band.

Our results on alpha-ERDs are consistent with the initial hypothesis that modulation over the hemisphere contralateral to stimulation has an opposite sign with respect to direct stimulation. Indeed, as previous studies have shown that anodal tDCS strengthens the motor imagery alpha-ERDs in the stimulated region [14–16,18–20,23], we suggest that the same stimulation has an opposing effect when applied over the contralateral side. An opposing effect of tDCS on ERD between hemispheres was also found in [21], while our results are in partial disagreement with those found in [15,23]. However, in both [15,23] the feedback was encoded into a left-right cursor movement, controlled by the difference in power between electrodes in opposite hemispheres during left versus right hand motor imagery, while we gave feedback on ERD in subject-specific electrodes and bands and in a single-hand motor imagery design. Furthermore, in [23] a different stimulation setup was tested (they used HD-tDCS). Overall, although both studies mentioned involve BCI-guided motor imagery with feedback, the differences in the experimental paradigms and setups may explain the discrepancy in the outcomes.

We hypothesize that enhanced activation of the left (dominant) motor cortex by means of anodal stimulation may have inhibited the right motor cortex, thus reducing the generation of ERD. Interhemispheric inhibition between motor cortices is a well-known effect [29], thought to be mediated by transcallosal connections [29,47,48]. Several studies have shown the possibility of modifying interhemispheric balance through noninvasive stimulation [32,49–51]. Nevertheless, whether or not tDCS is able to directly influence transcallosal connections is still up for debate [51–53]. Although our results seem to support this point of view, it cannot be excluded that interhemispheric modulation is mediated by subcortical structures. Indeed, the loops involved in the generation of alpha-ERDs are both cortico-cortical and thalamo-cortical [54] and it has recently been shown that tDCS can have effects on subcortical structures too, like the thalamus or the caudate nucleus [55]. Given these premises, it cannot be excluded that at least part of the long-distance effect between hemispheres is subcortical in nature. Finally, since we used a contralateral supraorbital reference, there could be a direct influence of the current flow between electrodes, which could marginally affect the ipsilateral circuits of the unstimulated hemisphere [55].

While we found reduced alpha-ERD generation by anodal stimulation, we did not find a symmetrical facilitating effect of cathodal stimulation. Our results indeed suggest that cathodal stimulation on one hemisphere does not influence the behavior of ERDs in the unstimulated one. However, anodal and cathodal stimulations have not always been found in the literature to have symmetrical effects [30,55]. To give an example, Notturno et al. showed that, while anodal stimulation increased movement-related alpha-ERDs in the stimulated motor cortex, neither sham nor cathodal stimulations had any effect [30]. We can comment that our results are in line with theirs, although mirrored to the other hemisphere. Overall, this asymmetry in the transmission of the tDCS stimulus may be supported by a model like the one in Ursino et al. [56]. Although not specific for interhemispheric communication, the model in [56] supports the hypothesis that it is not guaranteed that the transmission of information between two cortical areas is symmetrical with respect to the type of input (inhibitory or excitatory), due to the non-linear properties of the neuronal cortical circuits. Generally speaking, the development of models to interpret the tDCS-induced modulations on cortical rhythms and their transmission between functionally related areas, e.g. specifically between motor cortices, would be useful to improve our understanding of tDCS and to guide its application. For this purpose, a model like the one in Mangia et al [57], which already integrates interhemispheric connection between motor cortices and simulates the phenomenon of ERD/ERS induced by motor imagery, could be a good starting point to capture and interpret the additional role of tDCS.

To sum up, our results suggest that alpha-ERDs in the target motor cortex can be influenced by contralateral tDCS in a polarity-specific manner. In particular, while cathodal stimulation does not induce a global effect, anodal stimulation seems to reduce contralateral ERD.

### 4.2 tDCS effect on spectral power

The results discussed in the previous section suggest that contralateral tDCS is not applicable in the context of BCI training. However, a second, more general aim of this work was to contribute to the understanding of the impact of tDCS on EEG rhythms between hemispheres. This section, which discusses the results of power spectral analysis, complements and completes the previous discussion on ERDs, by showing that not only anodal but also cathodal stimulation can affect contralateral EEG rhythms.

In line with previous research involving the stimulation of motor-related areas [30,58,59], we found that tDCS mainly influences the power in theta and alpha bands.

In regard to the theta band, we generally found power increments occurring during stimulation, regardless of stimulation type and task condition. However, since the effects tended to disappear after stimulation (Fig 5) and given the localization mainly close to the stimulation sites (C3 and Fp2), we suggest that this is a direct current effect. For the same reasons, we suggest that the alpha-power increase occurs during cathodal stimulation over central electrodes of the same type. This interpretation is in line with previous works [17,22,24] describing the tDCS artifact as a low-frequency power increase in the electrodes near the stimulation sites. Notably, the higher frequency component of the disturbance may be due to ongoing small voltage shifts of the stimulator to maintain a constant current despite the little changes in electrode-skin impedances [22].

In regard to more distant electrodes, the alpha-band spectral power analysis interestingly revealed both a polarity-specific and, in the anodal group, a task-specific effect of stimulation. Overall, we found that cathodal stimulation decreased contralateral alpha power, while anodal stimulation tended to increase it. Moreover, in the anodal group the power increase only appeared during motor imagery, leaving the reference condition unaffected.

The EEG rhythms in the alpha band have historically been considered resting rhythms for the brain [54,60] or, more recently, related to active inhibition and timing of processes [61]. In the sensorimotor cortex, it has been suggested that the alpha rhythm reflects the cortico-thalamic idling rhythm, when no somatosensory input is processed and no motor output is generated [60]. As the alpha rhythm desynchronizes with movement [62], reflecting activation of the area, synchronized alpha activity has also been related to active inhibition (for example, in situations where a response must be avoided, or non-related task areas have to be silenced) [61]. Given these premises, the widespread increase in alpha power seen in the post-anodal phase during motor imagery can be interpreted as a more inhibited state of the right motor cortex. We further hypothesize that this increase only manifests itself when the motor cortex is actively recruited, i.e. only during motor imagery, which explains why the “reference” state is unaltered. This behavior is in line with the findings of Notturno et al. [30], suggesting that only cathodal stimulation influenced the pre-trial resting condition, while it was unaffected by anodal stimulation.

With respect to ERD analysis, spectral power analysis gives some additional insights into the effect of cathodal stimulation also. Indeed, if the ERD outcomes indicate that cathodal stimulation does not affect the contralateral side, the latter analysis suggests that not only anodal, but also cathodal stimulation has long-distance effects, which is in line with previous research [51,59]. Overall, cathodal stimulation was linked to a decrease in alpha power in the right hemisphere, which may be interpreted as enhanced activation. However, although slighter, we found a similar effect in the beta bands. Furthermore, the effects were not task-dependent in this case, i.e. they were not altered by motor cortex recruitment during motor imagery, so the results should be interpreted with caution.

A final interesting result of spectral power analysis concerns the overall continuity of the effects during and after stimulation. If we exclude the spectral power increase observed in the theta band and in the central electrodes of the alpha band in the cathodal group, which we previously ascribed to a direct current effect, we can indeed observe that the activation pattern in the *post* condition is generally the same as in the *during* condition. This result is in line with the work of Mangia et al. in [28], indicating that the tDCS-induced alterations begin in the very first minutes of stimulation.

Altogether, spectral power results corroborate the hypothesis that anodal stimulation of the left motor cortex increases inhibition of the contralateral one. Indeed, the widespread increase in alpha power, reflecting a more inhibited state, only manifests itself when the motor cortex is actively recruited during motor imagery. In regard to the cathodal group, power data analyses add the information that not only anodal, but also cathodal stimulation has a long-distance effect, although it does not appear to influence ERD generation.

### 4.3 Limitations of the study and generalizability to a patient population

One potential limitation of the study design is the presence of feedback, which has introduced a familiarization effect whereby the investigated variable, the ERD, was not stable over time. Also, the reinforcement of each participant in a slightly different band by selection of the subject-specific frequencies with spontaneous SMR modulation could have increased the variability of the data. On the other hand, feedback is important to keep the participant engaged and motivated, and its absence does not guarantee stabilization of ERDs, as boredom and lack of concentration can occur after a while and affect performance. In addition, as the particular aim of this study was to test the applicability of the setup with respect to its rehabilitative potential, we preferred using the neurofeedback-guided motor imagery training paradigm as a starting point, to evaluate the adjunctive role of stimulation. Even though both the within- and between-subject learning effects were taken into consideration when interpreting and discussing the results, it is possible that a different setup, e.g. with or without different feedback, could have led to slightly different results.

A final point we would like to discuss is the generalizability of our findings to a patient population. In particular, as this was a pilot study on healthy controls, we suggest it is not entirely correct to conclude that the same null-effect would appear in a patient population. First of all, a possible ceiling effect in SMR control could have manifested itself in healthy users. Furthermore, differences in brain physiology, e.g. the interhemispheric imbalance after stroke [5], or the plastic reorganization of the brain, could modify the outcomes when translating to patients. For example, interhemispheric imbalance in stroke patients might result in contralateral cathodal tDCS having a beneficial effect on ERDs of the unstimulated hemisphere, as has been seen in motor recovery [5], by inducing relief of inhibition exerted by the contralesional side, not evident in healthy subjects. Therefore, although our findings would suggest not following the contralateral cathodal tDCS approach in the context of neurofeedback-guided motor imagery training, it cannot be excluded that a similar experimentation in stroke patients might lead to a slightly different outcome.

## 5 Conclusion

Both spectral power and ERD analyses suggest that anodal tDCS of one motor cortex results in inhibition of the contralateral one. Assuming the effect of anodal tDCS to be excitatory in the stimulated cortex, this outcome would confirm our initial hypotheses that: i) the ERDs on the target motor cortex may be modulated by contralateral anodal stimulation and ii) this modulation has an opposite sign with respect to the stimulated hemisphere (which is in line with the phenomenon of interhemispheric inhibition).

Unfortunately, we did not find a symmetrical ERD enhancing effect through contralateral cathodal stimulation, which suggests that this setup is not applicable in the rehabilitation context (although differences in brain physiology and especially interhemispheric imbalance might lead to a non-null effect in the stroke patient population). Nevertheless, spectral power results suggest that not only anodal, but also cathodal stimulation can induce long-distance effects on the contralateral motor cortex. Altogether, our results support some recent findings, as in [30], indicating the possibility of tDCS modulation being transmitted between functionally related cortical areas. We further suggest that the development of models to interpret the tDCS-induced modulations on cortical rhythms would be useful to improve understanding of the neuromodulatory effects of the technique and to guide its application.

## Supporting information

S1 Table Electrodes, bands and weights used for each subject for the composition of the control signal. The table shows the electrodes, bands and weights chosen for each subject after calibration for the composition of the control signal. The chosen locations and bands reflect the spontaneous SMR modulation of each participant.
(PDF)

S2 Table Results of ANOVA tests for spectral power analysis. The table shows the complete results of the multiway ANOVA tests performed on spectral power in the two groups (cathodal and anodal) and conditions (rest or motor imagery). The factors included in the analysis were *time*, *stimulation*, *frequency band*, *subject* (between-subject factors) and *electrodes* (within-subject factor).
(PDF)
