# Supplementary material for: Single-session tDCS over the dominant hemisphere affects contralateral spectral EEG power, but does not enhance neurofeedback-guided event-related desynchronization of the non-dominant hemisphere's sensorimotor rhythm
Source: PLoS One. 2018 Mar 7;13(3):e0193004. doi: 10.1371/journal.pone.0193004 (PMC5841755; doi:10.1371/journal.pone.0193004)
Supplement: S1 Table — The table shows the electrodes, bands and weights chosen for each subject after calibration for the composition of the control signal. The chosen locations and bands reflect the spontaneous SMR modulation of each participant. (PDF) [file pone.0193004.s001.pdf]

S1 Table. Electrodes, bands and weights used for each subject for the composition of the control signal. The table shows the electrodes, bands and weights chosen for each subject after calibration for the composition of the control signal. The chosen locations and bands reflect the spontaneous SMR modulation of each participant.

CATHODAL				ANODAL			
**Subject**	**Bands**	**Electrodes**	**Weights**	**Subject**	**Bands**	**Electrodes**	**Weights**
S_1	[8 10]Hz	Cp2	0.5	S_1	[8 10]Hz	Cp2	0.6
	[8 10]Hz	Cp4	0.5		[8 10]Hz	Cp4	0.4
S_2	[10 12]Hz	C2	0.5	S_2	[10 12]Hz	Cp4	0.5
	[10 12]Hz	C4	0.5		[12 14]Hz	Cp6	0.5
S_3	[12 14]Hz	C4	0.5	S_3	[10 12]Hz	C4	0.5
	[10 12]Hz	Cp4	0.5		[10 12]Hz	Cp4	0.5
S_4	[12 14]Hz	C4	0.5	S_4	[10 12]Hz	C4	0.5
	[12 14]Hz	Cp4	0.5		[10 12]Hz	Cp4	0.5
S_5	[12 14]Hz	Cp4	0.5	S_5	[10 12]Hz	Cpz	0.4
	[14 16]Hz	Cp6	0.5		[10 12]Hz	Cp2	0.6
S_6	[14 16]Hz	Fc4	0.5	S_6	[14 16]Hz	C2	0.6
	[14 16]Hz	Fc6	0.5		[14 16]Hz	C4	0.4
S_7	[16 18]Hz	Cp2	0.5	S_7	[10 12]Hz	Cp2	0.4
	[16 18]Hz	Cp4	0.5		[10 12]Hz	Cp4	0.6
S_8	[18 20]Hz	C4	0.4	S_8	[12 14]Hz	Cp2	0.4
	[18 20]Hz	Cp4	0.6		[12 14]Hz	Cp4	0.6
S_9	[20 22]Hz	C4	0.5	S_9	[18 20]Hz	C2	0.5
	[20 22]Hz	Cp4	0.5		[18 20]Hz	C4	0.5
S_{10}	[22 24]Hz	Cpz	0.5	S_{10}	[22 24]Hz	C2	0.5
	[22 24]Hz	Cp2	0.5		[22 24]Hz	C4	0.5
