# Supplementary material for: Single-session tDCS over the dominant hemisphere affects contralateral spectral EEG power, but does not enhance neurofeedback-guided event-related desynchronization of the non-dominant hemisphere's sensorimotor rhythm
Source: PLoS One. 2018 Mar 7;13(3):e0193004. doi: 10.1371/journal.pone.0193004 (PMC5841755; doi:10.1371/journal.pone.0193004)
Supplement: S2 Table — The table shows the complete results of the multiway ANOVA tests performed on spectral power in the two groups (cathodal and anodal) and conditions (rest or motor imagery). The factors included in the analysis were time, stimulation, frequency band, subject (between-subject factors) and electrodes (within-subject factor). (PDF) [file pone.0193004.s002.pdf]

S2 Table. Results of ANOVA tests for spectral power analysis. The table shows the complete results of the multiway ANOVA tests performed on spectral power in the two groups (cathodal and anodal) and conditions (rest or motor imagery). The factors included in the analysis were *time*, *stimulation*, *frequency band*, *subject* (between-subject factors) and *electrodes* (within-subject factor).

**CATHODAL**				**ANODAL**			
**condition: “reference”**				**condition: “reference”**			
**Factor**	**F-value**	**df**	**p-value**	**Factor**	**F-value**	**df**	**p-value**
Time	49.9	2	$2.7 \cdot 10^{-22}$	*Time*	8.81	2	$1.5 \cdot 10^{-4}$
Stimulation	0.79	1	0.37	*Stimulation*	27.3	1	$1.7 \cdot 10^{-7}$
Frequency band	88.1	3	$2.7 \cdot 10^{-56}$	*Frequency band*	3.74	3	0.011
Subject	117	9	$1.3 \cdot 10^{-109}$	*Subject*	22.2	9	$1.7 \cdot 10^{-38}$
Electrodes	41.3	11	$2.9 \cdot 10^{-90}$	*Electrodes*	9.45	11	$2.9 \cdot 10^{-17}$
Time×stimulation	0.34	2	0.72	*Time×stimulation*	21.7	2	$3.7 \cdot 10^{-10}$
**condition: “motor imagery”**				**condition: “motor imagery”**			
**Factor**	**F-value**	**df**	**p-value**	**Factor**	**F-value**	**df**	**p-value**
Time	62.6	2	$9.3 \cdot 10^{-28}$	*Time*	26.6	2	$1.9 \cdot 10^{-13}$
Stimulation	1.98	1	0.16	*Stimulation*	90.2	1	$1.9 \cdot 10^{-21}$
Frequency band	157	3	$2.2 \cdot 10^{-99}$	*Frequency band*	30.2	3	$2.0 \cdot 10^{-19}$
Subject	237	9	0	*Subject*	33.5	9	$1.0 \cdot 10^{-58}$
Electrodes	109	11	$1.4 \cdot 10^{-249}$	*Electrodes*	64.3	11	$4.1 \cdot 10^{-144}$
Time×stimulation	3.52	2	0.029	*Time×stimulation*	26.5	2	$7.2 \cdot 10^{-14}$
